# Biomarker Metabolites Discriminate between Physiological States of Field, Cave and White-nose Syndrome Diseased Bats

**DOI:** 10.3390/s22031031

**Published:** 2022-01-28

**Authors:** Anna C. Doty, A. Dan Wilson, Lisa B. Forse, Thomas S. Risch

**Affiliations:** 1 Department of Biology, California State University Bakersfield, Bakersfield, CA 93311, USA; 2 Department of Biological Sciences, Arkansas State University, Jonesboro, AR 72467, USA; trisch@astate.edu; 3 Pathology Department, Southern Hardwoods Laboratory, Center for Forest Genetics & Ecosystems Biology, Southern Research Station, USDA Forest Service, 432 Stoneville Road, Stoneville, MS 38776, USA; alphus.wilson@usda.gov (A.D.W.); lbforse@gmail.com (L.B.F.); 4 Arkansas Biosciences Institute, Arkansas State University, Jonesboro, AR 72467, USA

**Keywords:** chiroptera, white-nose syndrome, disease biomarkers, early disease detection, electronic nose, healthy biomarkers, metabolomics, volatile organic compounds, VOCs

## Abstract

Analysis of volatile organic compound (VOC) emissions using electronic-nose (e-nose) devices has shown promise for early detection of white-nose syndrome (WNS) in bats. Tricolored bats, *Perimyotis subflavus*, from three separate sampling groups defined by environmental conditions, levels of physical activity, and WNS-disease status were captured temporarily for collection of VOC emissions to determine relationships between these combinations of factors and physiological states, *Pseudogymnoascus destructans* (Pd)-infection status, and metabolic conditions. Physiologically active (non-torpid) healthy individuals were captured outside of caves in Arkansas and Louisiana. In addition, healthy and WNS-diseased torpid bats were sampled within caves in Arkansas. Whole-body VOC emissions from bats were collected using portable air-collection and sampling-chamber devices in tandem. Electronic aroma-detection data using three-dimensional Principal Component Analysis provided strong evidence that the three groups of bats had significantly different e-nose aroma signatures, indicative of different VOC profiles. This was confirmed by differences in peak numbers, peak areas, and tentative chemical identities indicated by chromatograms from dual-column GC-analyses. The numbers and quantities of VOCs present in whole-body emissions from physiologically active healthy field bats were significantly greater than those of torpid healthy and diseased cave bats. Specific VOCs were identified as chemical biomarkers of healthy and diseased states, environmental conditions (outside and inside of caves), and levels of physiological activity. These results suggest that GC/E-nose dual-technologies based on VOC-detection and analyses of physiological states, provide noninvasive alternative means for early assessments of Pd-infection, WNS-disease status, and other physiological states.

## 1. Introduction

White-nose syndrome (WNS), a necrotrophic fungal disease found primarily in cave-dwelling Nearctic bats, has reduced bat populations in some eastern regions of North America by as much as 99% [1,2,3]. WNS is caused by a keratinophilic fungal pathogen, *Pseudogymnoascus destructans* (Pd), that develops epidermal and deep, subcutaneous skin infections in at least twelve insectivorous bat species [4,5]. The disease was first discovered in North American bat populations in 2006 (within New York caves) and is believed to have originated from Europe where bats have likely coexisted with *P. destructans* for millenia [6,7]. Skin-infections by *P. destructans* result in characteristic epidermal erosions that develop into necrotic flecks, filled with fungal hyphae on the muzzles, ears, and wings. Major Pd damage to wing tissues is most significant because it interferes with gas exchange, adjustment of blood flow, maintenance of body temperature, initiation of hibernation, and reduces flight capabilities [4]. In severe cases, necrotic areas coalesce to form larger dark necrotic zones or wing patches that may fall away in advanced stages, leaving vacant holes in the wing. White mycelium of the fungus may eventually aggregate to cover most of the bat skin surface area and appear as white plumes [8]. WNS systemically disrupts the normal physiology of hibernating bats, leading to adverse physiological cascades, including chronic respiratory acidosis, frequent fat-depleting arousals causing starvation and dehydration, Immune Reconstitution Inflammatory Syndrome (IRIS) during conscious periods of euthermia (following hibernation), and ultimately death [9,10,11,12,13].

The precipitous decline in tricolored bat (*Perimyotis subflavus*) populations in North America, due to the widespread devastating effects of WNS since 2016, has resulted in this species being nearly extirpated from several U.S. states. Cheng et al. [5] estimated the scope of threat of WNS to *P. subflavus* was extreme and indicated that 59% of the geographical range of this species was affected, based on the IUCN range map, and 93% of the population declined in its range within seven years after WNS was first detected. The high vulnerability of *P. subflavus* to population declines has been attributed to low reproductive rates, high susceptibility, relatively small size, and low tolerance to Pd infections at relatively low inoculum loads compared with larger bats. Consequently, this species is currently rated as a vulnerable species, but is being considered for endangered species status by the International Union for the Conservation of Nature (IUCN) [14].

Previous research has investigated various aspects and means by which WNS affects bats physiologically. However, a major gap in knowledge has been the lack of information and methods for measuring changes in bat physiology associated with pathogenesis, necessary for monitoring disease development noninvasively over time. One method for gaining a deeper understanding of physiological effects of WNS on bats and monitoring these physiological effects is through detecting and monitoring emissions of volatile organic compounds (VOCs) from bats in different physiological states and levels of activity. The presence or absence of specific VOCs and increases or decreases in concentrations of specific VOC emissions reveal different physiological states of bats based on their state of health and activity that change over time. Unique changes in VOC emissions from animals, associated with pathogenesis of specific diseases, usually are due to disruptions in normal host metabolic pathways [15,16]. Monitoring changes in VOC emissions, derived from appropriate biological sample sources, is a useful means for detecting changes in host physiological processes caused by disease which may otherwise be undetected at early stages using traditional diagnostic methods. Detectable changes in VOCs may result from host–pathogen interactions, inflammatory responses, or even direct tissue damage or injury. Prior research has shown that altered VOC composition or concentrations (metabolomic changes) are effective indicators of physiological disruption in animals; including evidence of disease states such as sepsis, and physiological stresses associated with lung injury, hemorrhagic shock, ketosis, starvation, and inflammation [17,18,19,20,21].

VOC-emission analysis has been used to determine the presence of disease states and for diagnosis of a variety of domestic and wild animal diseases including tuberculosis, caused by *Mycobacterium bovis* infections in cattle, white-tailed deer, and wild boar (*Sus scrofa*) using exhaled breath and fecal samples [22,23,24,25,26]. The causal agent of paratuberculosis in goats, *M. avium* subsp. *paratuberculosis*, has been successfully detected for diagnosis of this disease by analysis of VOC emissions from exhaled breath and feces [27,28]. Infection by *Brucella abortus*, causing brucellosis in bison, has been explored through analysis of VOCs from exhaled breath, indicating potential feasibility for disease diagnosis [29]. Diagnosis of the transmissible spongiform encephalopathy Chronic Wasting Disease (CWD) in white-tailed deer using feces-derived VOCs also has shown promise [30]. The success in diagnosing disease-states in vertebrates through VOC analysis encourages wider application, particularly as a non-invasive tool for wildlife disease monitoring, especially for threatened or declining populations, including certain bat species.

Electronic-nose (e-nose) devices, gas-sensing instruments capable of detecting specific mixtures of VOCs in air samples by aroma signature patterns, also show potential for noninvasive early detection of human diseases as well as WNS in bats [31]. Previous research has demonstrated that the portable C-320 e-nose, containing a carbon black polymer composite (CBPC) 32-sensor array, effectively distinguished between nine species of North American bats based on analysis of bat-derived VOC-metabolite emissions [32]. Other e-nose instruments, such as the Alpha MOS Heracles GC/E-nose with dual-technology VOC detection and GC chemical-analysis, has provided a novel means for early detection of WNS in bats prior to bats showing visible symptoms, reducing the need for tactile disturbance using qPCR swabs which are less reliable for early WNS detections. Use of an e-nose in conjunction with supporting VOC analysis shows promise for taking advantage of early WNS disease-detection capabilities to potentially provide swift, timely application of effective treatments prior to the onset of devastating symptoms. Disease prognoses are significantly improved by implementation of early, interventional disease-control treatments at early stages of disease development [33].

Previous investigations involving VOC-associated research in connection with WNS have been limited. The potential use of certain VOCs derived from microbes to fumigate caves was investigated to help control the growth and conidial sporulation of *P. destructans* to help reduce inoculum loads and Pd-infections [34,35,36,37]. Additional research indicated that the metabolic VOC profile of *P. destructans* differed significantly from those of other *Pseudogymnoascus* species [38]. However, very little research has focused on innate VOCs present in healthy and diseased bats. Knowing the compositional differences of VOCs in healthy vs. diseased bats will yield greater insight into specific physiological effects of WNS on bats which result in measurable changes in VOC whole-body emissions.

Other potential applications of VOC-emission analysis, besides detecting disease states, may include insights into defining normal physiological states of organisms at different levels of physical activities and under different environmental conditions. For example, VOC profiles of human skin cells differed based on culture conditions [39], and VOC profiles of exhaled breath in humans are affected by exercise [40]. Torpor and hibernation are important physiological states noted in heterothermic endotherms whereby metabolic rate and body temperature are markedly reduced for short or prolonged periods of time. Because bats are heterothermic endotherms, it is possible that their VOC profiles are markedly different when in active and inactive states. Bats exhibit a wide suite of physiological changes that occur as they transition from active to torpid states for hibernation [41]. Very little research has focused on determining VOC chemical biomarkers associated with differing physiological states in bats. Most research in this area for small mammals has focused on changes in internal metabolites of the thirteen-lined ground squirrel (*Spermophilus tridecemlineatus*). For example, this species expressed differing concentrations of glucose, lactate, alanine, succinate, β-hydroxybutyrate, glutamine, and betaine in the liver based on the animal’s state of torpor or activity [42]. Another study identified 106 blood plasma-derived metabolites that significantly differed in composition based on the state of torpor in *S. tridecemlineatus* [43]. To our knowledge, no previous research has analyzed VOC emissions in external air samples to identify volatile chemical biomarkers associated with heterothermy or disease in any mammalian hibernator. Because WNS is most detrimental to bats during hibernation [13,44,45], identification of chemical biomarkers and VOC e-nose profiles for differentiating diseased hibernating bats from those of healthy hibernating and healthy active bats is crucial for developing effective and measurable real-time indicators for monitoring healthy vs. disease physiological states.

The analysis and identification of specific metabolites that could serve as potential chemical biomarkers for early detection of physiological disorders and disease has been used extensively as a model approach in numerous biomedical and metabolomic studies [46,47,48]. Metabolites identified as possible disease biomarkers include those derived from the pathogen (for biotic diseases), including some unique virulence factors, and metabolites that form due to pathogenesis that causes disruptions of normal metabolic pathways in the host. Metabolomic biomarkers are metabolites that are produced in higher or lower quantities than levels normally found in healthy individuals, usually in association with specific metabolic pathways [49]. The specificity of effects of pathogens on certain metabolic pathways of a host is commonly a diagnostic characteristic of specific pathogen groups [16]. Other metabolites useful as chemical biomarkers for detecting the presence of disease include certain microbial metabolites, produced by abnormal gut microbes, which displace or modify the healthy gut microbiome, due to disease processes such as in microbial dysbiosis [50]. By extension, metabolite biomarkers also are useful, as a portion of the total metabolome (entirety of metabolites produced by an organism), for detecting different pathophysiological states (pathogenesis) within an organism or diversions in physiological processes caused by changes in environmental conditions that induce hormone-mediated cascadic modifications in many metabolic pathways (e.g., initiation of torpor for hibernation) [51].

The aims of this study were to (1) determine differences in VOC composition of whole-body emissions from tricolored bats in three categories, including (a) healthy, active bats in summer; (b) healthy, torpid bats in winter, and (c) diseased, torpid bats affected by WNS; (2) identify unique chemical VOC biomarkers indicative of specific types of physiological activities and states; and (3) identify differences in overall VOC profiles of these three sample groups based on electronic-nose PCA aroma-map comparisons. For our working hypothesis, we predicted that all three groups of *P. subflavus* bats would exhibit different VOC composition due to their differing physiological states and that we would find specific VOC biomarkers associated with the WNS-disease state as well as other biomarkers specifically associated with different levels of bat activity.

## 2. Materials and Methods

### 2.1. Bat Sampling Locations, Timing, and Procedures

Bats were hand-captured in February–March 2018 from two caves located in the Ozark-St. Francis National Forest in Arkansas (names of caves withheld to reduce visitation traffic; henceforth shall be referred to as “Cave A” and “Cave B”) during daylight hours while bats were torpid. Cave A contained known populations of tricolored bats (*Perimyotis subflavus*) exhibiting characteristic visible symptoms of WNS (epidermal erosions filled with fungal mycelium on the muzzles, ears, and/or wings) while Cave B contained *P. subflavus* without characteristic visible symptoms of WNS. Because the metabolite expression of WNS differs based on infection status (i.e., visible fungal growth vs. early-stage Pd-infection), and that WNS is typified by visible symptoms in late winter, we considered bats from Cave B to be disease-free. We collected 24 cave air samples from *P. subflavus* of known sex from the caves (Cave A: n = 9, N = 1F, N = 8M; Cave B: n = 15, N = 7F, N = 8M).

Additional bat air samples were collected in January–March of 2017 and 2018 in the Ozark-St. Francis National Forest and on local private properties (specific locations withheld to reduce visitation). These samples were obtained from undisturbed (not handled) bats on cave walls. We collected 20 cave air samples from *P. subflavus*, including 10 torpid healthy bats (from 6 different caves) and 10 torpid WNS-diseased bats (from 5 different caves), to serve as controls in determining the effects of handling bats only for biomarker-identification analysis. Some differences in the temperature and humidity profiles of field sites were evident; winter caves had minor variation in temperature (mean 13.6 ± 3.5 °C with greater variation in relative humidity (mean 74.2 ± 11.6%) and summer field sites were warmer (mean 26.8 ± 0.3 °C) than winter cave sites (humidity information unavailable).

We collected four field bat air samples in July–August 2017 from healthy and active summer bats, (N = 2F, N = 2M). Bats were captured using mist nets (Avinet, Portland, ME, USA) set at dusk in forested landscapes in Craighead Co., Arkansas, and Grant and Natchitoches Parishes, Louisiana, respectively, as reported previously [32]. To reduce potential of Pd cross-contamination, latex gloves were discarded after handling each bat. Containers (jars) and holding bags were used once for each bat and autoclaved or sanitized the following day for re-use. Any instruments that required re-use during the same evening (e.g., scale, calipers) were sanitized between measurements.

### 2.2. Ethical Considerations

All activities were conducted in accordance with the Institutional Animal Care and Use Committee (IACUC) regulations at Arkansas State University (document IACUC # FY16-17-22), using research permit approvals for bat captures from Arkansas Game and Fish Commission (Permit # 051020161) and from Louisiana Department of Wildlife and Fisheries (Permit # LNHP-17–024).

### 2.3. Bat Air-Sample Collection

Whole-body air samples were collected from bats expressing different physiological states at locations outside and inside of caves. Healthy torpid cave bats were collected from cave walls, during the winter hibernation season, from individuals that were asymptomatic and apparently healthy without WNS diagnostic symptoms (i.e., no skin lesions or signs of white fungal mycelium growing on muzzle and wings), and presumably with active immune systems. Torpid Pd-infected diseased bats with WNS symptoms were likewise captured during the same winter hibernation period. Healthy active field bats were captured during summer outside of caves. The bat air-sampling procedure utilized was previously outlined in some detail [32], but with some modifications as presented below using a different air-collection device.

The design and components of the glass sampling chambers used for collection of VOCs from bats were described previously [32]. Following capture, bats were held for ~10 min to isolate bats from ambient air and create headspace for sampling. To minimize stress, cloth bags were placed over the sampling chamber during the isolation period. After the 10 min isolation period, the vacuum-pump box was switched on and the VOC air sample bag inside of the vacuum box was filled with air from the sampling chamber at a rate of 0.5 L/min. The ultra-zero pure air bag (connected to port 2 of the air sampling chamber) replaced the air removed from the sampling chamber at the same rate to prevent ambient air contamination and to equalize pressure within the sampling chamber.

**Figure 1:**
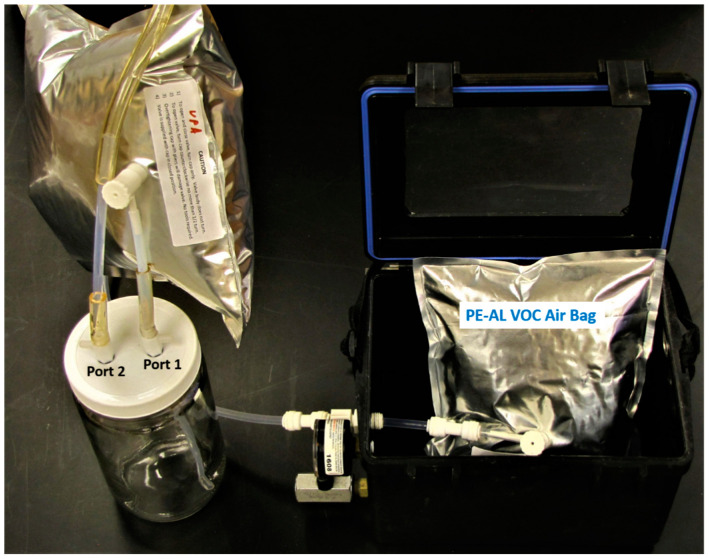
Bat VOC air-collection apparatus assembly. Xitech vacuum chamber (at right, containing PE-AL VOC air-sampling bag inside) with glass bat air-sampling chamber (at left) connected via two port valves to FEP tubing with Port 1 (receiving air inflow from pure zero-air replacement bag), and Port 2 (allowing outflow of bat sample air to input port of Xitech air-collection chamber and internal VOC air-collection bag).

The bat VOC-sampling chamber was connected to a Low Vac 1 L vacuum box air sampler (Model 1060, Xitech Instruments, Inc., Placitas, NM, USA), containing an identical PE-AL VOC air-sample bag inside, and to a 1 L pure air replacement bag through separate FEP tubing connections to Ports 1 and 2 as described previously [32]. The complete bat VOC air-collection apparatus assembly with FEP tubing connections, air-transfer ports, and PE-AL air bag with orientations is shown in Figure 1.

Explanations behind the methods and rationale for the air-collection apparatus designs, developed for bat whole-body VOC-collection in this study, were provided previously along with cleaning procedures utilized prior to re-use of these devices [32].

Control air samples were taken from inactive (torpid) cave-wall bats, not handled or placed in VOC sampling jars, using elongated 4.8 mm ID, 6.4 mm OD FEP tubing, raised to close proximity of bats using a 7.6 m (length) non-conductive fiberglass telescoping pole (Hastings, Hastings, MI, USA), which was connected to the VOC air-collection device. The collection of bat air within the sampling tube was fractionated and collected in PE-AL VOC sampling bags on a time-delayed basis, depending on the length of the sampling tube used and the flow rate of the vacuum sampler, to avoid collecting air already present in the sampling tube prior to acquisition of air samples from bats. All bat air samples collected in the field and caves using PE-AL VOC-collection bags were transported overnight to the USDA Forest Service, Southern Hardwoods Laboratory (SHL, Stoneville, MS, USA) pathology laboratory for e-nose analyses.

Field-captured bats were handled according to methods used previously [32]. Cave-captured bats were carefully placed back on the cave wall in the same location. The entire procedure, from capture to release, generally did not exceed 20 min per bat.

The PE-AL air-sampling bags, containing bat air samples, were placed into styrofoam boxes, inserted into custom-fit cardboard boxes, and shipped by overnight mail to the Southern Hardwoods Laboratory, Pathology Department, Stoneville, MS. Shipping boxes were stored at 4 °C within a walk-in cool room until air samples were individually prepared for chemical analysis. Samples available for immediate analysis were maintained at 21 °C prior to preparation for analysis.

### 2.4. Pre-Analysis Bat Air-Sample Preparation

An analytical reference standard custom mixture (Restek, Bellefonte, PA, USA, product number 561203), composed of 11 sequential aliphatic alkanes (C7–C17), was utilized prior to all GC sample analyses to set up Kovats calibrations for determinations of Kovats Retention Indexes (KRI) for specific chromatographic peaks present in GC chromatograms.

### 2.5. GC/E-Nose Configuration Parameters and Data Acquisition

The details of methods (instrument configuration parameters, data acquisition, and procedures) utilized with the Heracles II GC/Electronic-nose system (Alpha MOS, Toulouse, France) for chemical analysis of headspace volatiles derived from bat whole-body air samples, were identical to those used previously for analysis of microbial headspace VOCs of the Pd-pathogen and related *Pseudogymnoascus* species [38].

### 2.6. GC Identification of VOC Components and Biomarkers

The Statistical analyses of e-nose, smellprint signatures, and principal component analysis (PCA) data were carried out using Alphasoft v14.20 and AroChembase software using methods described previously [38]. Individual peaks recorded in GC chromatograms were tentatively identified based on comparisons of Kovat values, calculated for each unknown bat VOC-component (KRI-t) present in bat air-sample headspace, with Kovat values (KRI-v) of known compounds within the Kovats Retention Index (KRI) reference library. These comparisons indicated potential identities of peak compounds, based on the nearest matches of KRI values from among >83,000 compounds present in the KRI reference library. In addition, Relevance Index (RI) values, indicating percentage probability of identity match, based on Kovats values for specific compounds, were displayed with GC-output data in association with KRI-t values of each tentative identity compound. Statistical differences in quantities of individual VOCs detected for metabolomic analyses were determined using SigmaPlot 14.5 (Inpixon, Palo Alto, CA, USA) with Kruskal–Wallis one-way ANOVA on ranks followed by Dunn’s tests at (*p* < 0.01).

Gas chromatographic data used in identifying chemical biomarkers associated with specific types of physiological activities of the three *P. subflavus* bat air-sample types were collected primarily from handled bats collected from cave walls that were placed within VOC-sampling chambers. The physiological biomarkers identified from bats removed from cave walls and placed in VOC-sampling chambers included healthy biomarkers, active field biomarkers, and bat-activity biomarkers. Some additional air samples, collected from undisturbed bats only on cave walls were collected as controls to identify metabolomic biomarkers, such as torpor biomarkers, only found in completely inactive, torpid bats that were not handled.

### 2.7. Principal Component Analysis of E-Nose Data

Three-dimensional PCA was performed on e-nose sensor-response data derived from whole-bat air samples to compare the relatedness between healthy and active field bats, torpid, nonsymptomatic (healthy) bats, and torpid, symptomatic WNS-diseased bats based on aroma signature patterns derived from e-nose sensor array output responses to VOC-metabolite mixtures in headspace. Data used in PCA comparisons of the three *P. subflavus* air sample types also included air samples collected from undisturbed bats on cave walls in addition to samples collected from handled bats placed within VOC-sampling chambers. These additional data of undisturbed cave-wall bats provided more evidence as controls to demonstrate the effective discrimination of air sample types from bats in natural hibernacula settings, not just in isolated, more controlled closed sampling chambers without access to ambient cave air. PCA mapping distances between plot centers of data clusters of each aroma class (bat-air sample type) were determined by pairwise comparisons of sample data in all possible combinations. In addition, Pattern Discrimination Index (PDI), expressed as a percentage difference between sample types, was calculated based on differences in aroma smellprint patterns. PDI-values provided approximations of statistical level of discrimination (*p*-values) between compared sample types.

## 3. Results

The output data from the dual-technology, Heracles II GC/E-nose instrument analysis of whole-body VOC emission provided two types of chemical data that were useful in characterizing the physiological states of *P. subflavus* bats under different physical environments and levels of physical activity. Data from the dual-column GC FID detector provided gas chromatographic data for identifying individual VOCs detected in the headspace of the bat air-sampling chamber. Additional chemical data from the e-nose sensor array yielded information specifying indications of chemical relatedness between VOCs present in air samples from the three *P. subflavus* sample types. Results from both chemical data types are presented in the following, separate subsections.

### 3.1. Dual-Column GC Analyses

**Figure 2:**
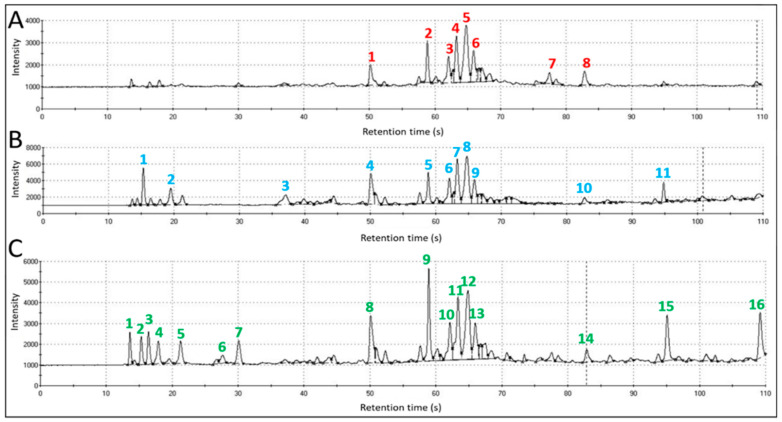
Gas chromatograms, derived from DB-5 column, displaying numbered major peaks detected in whole-body VOC emissions from *P. subflavus* bats. (**A**) Pd-infected cave bats = Pd-infected, WNS-symptomatic torpid bats with reduced physiologically activity, but with more frequent arousal episodes due to dermatophytic, Pd-associated irritation (**B**) Healthy cave bat = physically inactive, Pd-uninfected, torpid bats with greatly reduced physiological activity; and (**C**) Healthy field bats = physically active foraging bats with full-range of metabolic activities.

Fast-gas chromatographic analysis of headspace volatiles derived from bat whole-body air samples of three types (healthy active field, torpid healthy cave, and torpid Pd-infected cave bats) indicated significantly different numbers, molecular weights, and quantities of VOCs, distinguishing the composition of VOC emissions from bats sampled from different environments and having different physiological states. A comparison of gas chromatograms, derived from the DB-5 column for each sample type, is provided with numberings of major GC-peaks in Figure 2A–C. Differences in VOC profiles of each sample type are demonstrated by vastly different patterns and distributions of chromatographic peaks, peak areas, and retention times (RTs). The smallest number of major VOC peaks was recorded for air samples from Pd-infected bats with WNS. All eight major peaks in the chromatogram from diseased cave bats were clustered within a narrow range of 50–85 s RTs, containing medium-sized VOCs in the molecular weight range of approximately 124–205 daltons (Figure 2A). The distribution of the largest eleven peaks of headspace volatiles in the chromatogram for air samples from healthy torpid cave bats occurred within a wider range of 15–95 d RTs, having a molecular weight range of 75–234 daltons (Figure 2B). The widest distribution range and number of major VOC peaks was recorded for healthy, active field bats with RTs ranging from 13–110 s, and a wider molecular weight range of 72–280 daltons (Figure 2C). In all three bat sample types, the largest peaks (with greatest peak areas) occurred in the mid-range of VOC molecular weights within the 50–67 s RTs range using the DB-5 capillary column. Torpid bats, regardless of Pd-infection status, exhibited lower numbers of major VOC peaks relative to physically active field bats.

### 3.2. Chemical Analyses of Bat VOCs

The tentative chemical identities of VOCs detected in whole-body air samples obtained from *P. subflavus* individuals (of three sample types) were determined by use of RTs in combination with supporting reference data from the 83K+ compound KRI reference library, compared with Kovats values established using 11-alkane reference standards, and Relevance Index (RI) values, indicating percentage probability of identity matches with known compounds. The tentative identities of best-match VOCs were selected (from a list of possible matches) based on compounds for which the highest RI ranges (generally >90% match at the top of the range) were calculated using data from both DB-5 and DB-1701 GC columns.

Peaks on GC chromatograms were designated as major or minor peaks with different peak area ranges that varied depending on sample type. Major peaks were defined for each sample type to include all peaks having the following approximate peak area ranges: 120–4600 for active healthy field bats, 380–4400 for torpid healthy cave bats, and 220–1900 for torpid Pd-infected cave bats. All peaks with peak areas having less than the minimum values, defined for the ranges of major peaks for each sample type, were considered minor peaks.

The VOC profiles of active, healthy field bats, sampled outside of caves, consisted of 16 major peaks on GC chromatograms with a wide range of RTs and representing 10 chemical classes (Table 1). Alkanes (25%) and ketones (19%) were the most frequent VOC chemical classes represented by this sample type. On a peak area basis, the largest VOC peaks consisted of alkane, benzene derivatives, terpene, and ketone chemical classes in order of highest to lowest major peak area, respectively. Active field bats released three chemical classes of VOCs with major peak areas which were not found as major-emission VOCs from cave bats, either healthy or Pd-infected, including alcohols, amines, and terpenes.

The tentative identities of the largest major VOC peaks (by peak area) found in volatile emissions from active field bats potentially provided some indications of major metabolic pathways associated with physical activity (such as flying), physiological processes occurring in nontorpid states, and exposures to chemicals outside of the cave environment. Three alkanes, 5-ethylnonane (Peak 11), 2-methyldecane (peak 12) and 3-methyldecane (Peak 13), were the most abundant alkanes released from field bats. Heptane (peak 7) was a low molecular weight alkane (RT = 30.0), discovered with a minor peak area, which was only present in emissions from active healthy bats. The second most abundant major VOC (peak 9) was tentatively identified as phenol, classified as an alkane. A benzene alcohol, phenol (peak 9), exhibited the highest peak height (among all peaks in the health field-bat VOC profile) and occurred among the largest peak-area mid-range molecular weight VOCs at RT = 58.9. Limonene (peak 10), a cyclic monoterpene, is another major-peak volatile found in the VOC emissions of active bats. The other healthy field bat major-peak VOC was 3-mercapto-4-methyl-2-pentanone (peak 8), an unusual sulfur-containing ketone. The two largest molecular weight VOCs discovered in field bats was 2-pentadecanol (Peak 15), a long-chain secondary alcohol, and heptadecanal (peak 16), an aliphatic long-chain fatty aldehyde.

Healthy, torpid cave bats exhibited VOC profiles that were different from those of active, healthy field bats. Chromatograms of inactive, torpid cave bats contained 11 major peaks, over a wide range of RTs and molecular weights, but represented by only six chemical classes (Table 2). Alkanes were the most frequent chemical class represented among major peaks, accounting for 45% of total VOC emissions. The largest VOC peaks (on a peak area basis) consisted of alkane, ketone, and benzene alcohol, chemical classes from highest to lowest major peak area, respectively. Torpid cave bats released a single compound in the ether chemical class that were not found among major VOC-emissions from active field bats or Pd-infected. In addition, two major peaks not found among major VOC emissions from active field bats or Pd-infected bats include a unique alkane, 2-methylheptane (peak 3), and a lactone, δ-dodecalactone (peak 11).

Five alkanes, including 2-methyldecane (peak 7), 5-ethylnonane (peak 6), 3-methylheptane (peak 3), 3-methyldecane (peak 8), and undecane (peak 9) comprise the largest major VOC peaks (by peak area) found among volatile emissions from healthy torpid cave bats. The major alkane 3-methylheptane (peak 3, RT = 37.1) was unique to active field bats. The next most abundant VOC released from healthy cave bats was a ketone, 3-mercapto-4-methyl-2-pentanone (peak 4). Only a single aromatic compound, phenol (peak 5), a benzene alcohol, was found among major-peaks VOCs. The majority of the seven major-peak (by area) VOC emissions from torpid, healthy cave bats were medium molecular weight compounds (peaks 4–8) in the middle region (RTs = 50–67) of GC chromatograms. Two additional lower molecular weight, major-peak VOCs also were discovered, including a tertiary alkyl ether, t-butylmethylether (peak 2) and peak 3, described previously.

Symptomatic, WNS-diseased bats with noticeable Pd-infections and white mycelial masses on the skin surfaces near exposed areas of the muzzle and ears exhibited a significantly reduced VOC profile compared to healthy bats. Only 8 major peaks were observed in gas chromatograms of inactive, torpid infected cave bats. All peaks were medium molecular weight VOCs in a narrow wide range (RTs of 50–83) and represented by only five chemical classes, including alkanes, ketones, benzene alcohol, ester, and lactone (Table 3). Alkanes were the most frequent chemical class represented among major peaks, accounting for 50% of total VOC emissions. The largest VOC peaks (on a peak area basis) consisted of alkane, ketone, and benzene alcohol chemical classes from highest to lowest major peak area, respectively.

Four alkanes comprised the largest major VOC peaks (by peak area) found among volatile emissions from torpid WNS-diseased bats, with tentative identities of 2-methyldecane (peak 4), 5-ethylnonane (peak 3), 3-methyldecane (peak 5), and undecane (peak 6), from highest to lowest peak areas, respectively. Like healthy cave bats, WNS-diseased bat emissions contained undecane (peak 6), but at a mean level only about half the level found in healthy cave bats. The next most abundant VOC released from healthy cave bats was a ketone, 3-mercapto-4-methyl-2-pentanone (peak 1). A single benzene alcohol, phenol (peak 2), was the third most abundant VOC tentatively identified among the major-peak emissions in diseased bats.

A comparison of the total major and minor VOC emissions detected from the three sample types is summarized by chemical classes in Table 4. A total of 10 chemical classes of VOCs were tentatively identified among the major peaks of VOCs recorded in gas chromatograms of all sample types. These data provided indications of significant differences in VOC composition and physiological states by sample type.

The abundance and diversity of different major and minor VOCs present in whole-body emissions decreased in magnitude (from higher to lower) in healthy field bats, torpid healthy cave bats, and torpid WNS-diseased cave bats, respectively. Consequently, healthy cave bats emitted 14.9% fewer detectable total VOCs than healthy field bats, and torpid WNS-diseased bats emitted 25.8% fewer detectable total VOCs than torpid healthy cave bats. Thus, torpid WNS-diseased bats released 36.8% fewer detectable total VOC emissions than healthy field bats.

The composition (or diversity) of VOCs found in whole-body emissions from bats of the three sample types also varied considerably. Healthy field bats exhibited major VOC emissions consisting of compounds from at least 8 chemical classes, compared to 6 chemical classes represented in major VOC emissions from torpid healthy cave bats, and only 5 chemical classes represented in major VOC emissions from torpid WNS-diseased bats. All three sample types emitted major VOCs in common from four chemical classes including alkanes, benzene alcohols, and ketones, and lactones. Torpid healthy and torpid WNS-diseased bats had unique ketone (chemical class) major peak emissions that were absent in healthy field bats. A unique ether, 2-butylmethylether (peak 2) was only found in VOC emissions from torpid healthy cave bats, but not torpid diseased bats or healthy field bats. A specific lactone, δ-dodecalactone (peak 11), also was present only in torpid healthy cave bats, but not in heathy or diseased bats. Three alcohols were uniquely produced only by active field bats which included ethanol (peak 3), S(+)-2-butanol (peak 5), and 2-pentadecanol (peak 15). Torpid WNS-diseased bats produced an ester, hexyl pentanoate (peak 7) that was only found in Pd-infected samples.

Some additional manmade compounds from extra-cave sources, not derived from bat metabolic pathways, also were found among minor peak area volatile emissions from bats, particularly including different types and classes of agricultural pesticides. Pesticide VOC emissions were discovered at different levels in bats from all three sample types (Table 5). The highest levels of pesticide emissions, based on GC chromatogram peak areas, occurred in healthy, active field bats outside of caves, presumably having greater exposure to various pesticides in the environment. Lower quantities of pesticides were found in VOC emissions from inactive (torpid) cave bats. Pesticide levels in VOC emissions from healthy cave bats were consistently higher than in Pd-infected cave bats. Pesticides occurred less commonly in symptomatic WNS-diseased bats.

Most pesticides discovered among bat volatile emissions were insecticides and herbicides. The seven insecticides detected were tentatively identified as organophosphates, phosphorothioates, and organothiophosphates, which are effective acetylcholinesterase inhibitors used to kill agricultural insects and occasionally mite pests. Five herbicides were identified as triazine and thiocarbamate herbicides, having photosynthesis inhibitor activity and preemergence growth inhibitor modes of action, respectively. A single carbamate ester herbicide with selective growth inhibition properties to graminaceous weeds was also discovered. No other pesticide types were detected.

All pesticides detected among bat volatile emissions were predominantly higher molecular weight VOCs (RTs = 76–98) with relatively low volatility.

### 3.3. Identification of Chemical Biomarkers of Physiological States

Analysis of the specific chemical composition of VOC emissions detected in each sample type were used to help identify and characterize individual bat VOC metabolites as potential chemical biomarkers useful as indicators of bat physiological states or metabolic conditions depending on physical activity, environmental location, and WNS-disease (Pd-infection) status.

Biomarker VOC metabolites, tentatively identified within GC chromatograms derived from analysis of volatile emissions from healthy field, healthy cave, and Pd-infected bats, were found to be associated with specific types of bat physiological states (defined above) as summarized in Table 6. Thirteen total VOC biomarkers were detected as six low to moderate molecular weight compounds (peaks 1–6) with (RTs = 14–46), and seven higher molecular weight compounds (peaks 7–13), with (RTs = 48–105).

The VOC biomarkers identified in association with different physiological states were categorized into two major groups, referred to here as: (1) activity-specific biomarkers and (2) metabolomic biomarkers. The subcategories of VOC biomarkers identified within each of the two groups are specified, along with indications of detection within the VOC emissions of each sample type, within Table 7. To improve indications and comparisons of physiological states, biomarker data were collected from bats (removed from cave walls) and handled bats placed within VOC sampling jars, as well as from undisturbed wall (control) bats by which VOC emissions were taken in the very close vicinity of these bats to avoid handling disturbance.

Activity-specific biomarkers are VOCs metabolites that are only found in bats with certain specific and defined physiological states based on physical or metabolic activities. Two types of activity-specific biomarkers, including active-field (AF) biomarkers and torpor (T) biomarkers, were identified as those VOCs uniquely found in the volatile emissions from bats recently engaged in physical activity (flying, feeding, grooming, and other conscious activities) or bats primarily in an inactive and unconscious (torpid) state, respectively. Healthy field bats were the most consistent sample group that released AF-biomarker metabolites in their VOC emissions. AF-biomarkers were absent for WNS-diseased bats, whether handled or undisturbed, and from healthy cave bats handled during VOC sampling. Volatile emissions from undisturbed, healthy cave-wall bats only occasionally contained active-field biomarkers, presumably associated with intermittent consciousness or physical activity. Five AF-biomarkers were detected and tentatively identified as acetaldehyde, acetol, 2-methyhexane, 4-ethylhexadecane, and heptadecanal which are members of the aldehyde, ketone, alkanes, and aldehyde chemical classes, respectively.

Torpor biomarkers were always absent from the VOC emissions of healthy field bats but were consistently found in volatile emissions of all unconscious cave bats in torpid state, whether healthy or WNS-disease, and collected from handled or undisturbed bats during VOC sampling. Two torpor biomarkers were tentatively identified as 3-hexanol and ethyl cyclohexane from alcohol and cycloalkane chemical classes, respectively.

Metabolomic biomarkers are VOC metabolites produced and released at varying levels from bats of different defined physiological states. Two types of metabolomic biomarkers were detected, including healthy (H) biomarkers and conscious activity (CA) biomarkers. H-biomarkers are defined as volatile metabolites released in greater quantities within VOC emissions from healthy bats, whether from field or cave bats. By contrast, Pd-infected cave bats released either no H-biomarkers or significantly lower quantities of healthy biomarkers in their whole-body emissions than healthy bats (producing 2–4 times greater quantities). Four healthy-bat H-biomarkers were tentatively identified as propenal, 4-ethylheptane, glycerol, and γ-decalactone that are from aldehyde, alkane, polyol, and lactone chemical classes.

Another metabolomic biomarker type, referred to here as the conscious activity (CA) biomarkers, provided indications of consciousness or awake activity resulting from physiologically active states associated with either active field bats or episodes of consciousness occurring during periods of arousal between torpid states in cave bats. The emissions of CA-biomarker VOCs were highest in healthy field bats that were awake continuously (lacking torpid states). Cave bats with predominant torpid states with occasional episodes of arousal exhibited significantly lower levels of these VOCs. Healthy bats, handled during capture for sampling, showed similar levels of CA-biomarkers as undisturbed healthy wall bats. However, WNS-diseased bats, handled during capture, exhibited significantly higher levels of CA-biomarkers than undisturbed diseased wall bats. Two CA-biomarkers were identified as 2-(2-ethoxyethoxy) ethanol, ethyl octanoate, belonging to the alcohol (derivatives), and ester chemical classes.

### 3.4. Principal Component Analysis

**Figure 3:**
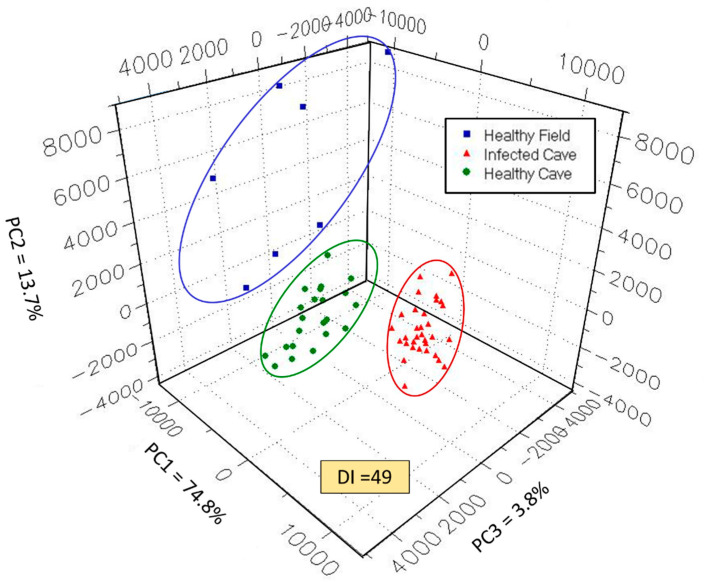
Principal component analysis e-nose aroma-plot of *P. subflavus* bat whole-body air VOC profile.

The multisensory output data from the Heracles II MOS e-nose sensor array were plotted to form an aromaplot for each of the three sample types. These data were analyzed by three-dimensional PCA to determine how well sample types were discriminated by principal components within VOC emissions and for determining the tightness of data clustering by plotted data points within each sample type or aroma class (Figure 3). VOC emissions from healthy field bats resulted in a wide range distribution of data points, indicating the greatest variability of VOC components in sampled air in field bats compared to cave bats. Data point clusters of plotted data for infected and healthy cave bats were considerably tighter with a narrower range of VOC composition relative to field bats. Discrimination Index (DI) indicates the relative strength of discrimination between all sample types included in the PCA test. This displayed discriminate index value (DI = 49), validated by Alphasoft V14.20 software, indicated a 3-d PCA test at *p* ≤ 0.01 level of significance in discriminating between sample types.

The percentages of total variance, accounting for the variability explained by each orthogonal principal component in the PCA, were as follows: PC 1 = 74.8%; PC 2 = 13.7%; and PC 3 = 3.8%. Thus, most of the variability in the PCA was accounted for by PC 1 (x-axis), whereas PC 2 (y-axis) and PC 3 (z-axis) accounted for only a minor proportion (17.5%) of the total data variance.

The chemical relatedness between VOC composition of whole-body volatile emissions from the three sample types (aroma classes) were determined using the quantitative statistical indicator, pattern discrimination index (PDI), by which the percentage differences in chemical relatedness between aroma classes were determined in all possible combinations. These PDI results are presented in Table 8. PCA distances indicate actual aromaplot distances between data cluster centers of aroma classes defined by PCA.

The biggest difference (77.1%) in chemical relatedness between headspace volatiles, indicated by PDI, occurred between Pd-infected cave bats and healthy cave bats. The next highest chemical difference (66.8%) in VOC composition was found between volatile emissions of healthy field bats and Pd-infected cave bats. The lowest percent difference (37.7%) in VOC emissions was found between healthy field bats and healthy cave bats.

Distances between plot centers of data clusters between aroma classes indicated similar results although plotting distance between data clusters of healthy field bats and Pd-infected cave bats was greater than plotting distance between Pd-infected bats and healthy cave bats. Nevertheless, these results are consistent in showing large differences in VOC composition of healthy bats from diseased bats regardless of location (inside or outside of the cave environment) and associated physiological states. Considerably more data were collected from cave bats, both healthy and Pd-infected, relative to field bats due to the difficulty of live captures. However, the data clustering for field bats was still relatively close despite the large diversity of ambient conditions of field capture sites because ambient air was removed and replaced with zero air prior building headspace for air sample collection.

Differences in PDI values, determined by pairwise comparisons between aroma classes, provided further confirmations of chemical data showing contrasts in VOC emissions, tentatively identified by GC analysis data (for each sample type), and a different measure of WNS-disease effects on alterations of bat physiology in the change from healthy to WNS-disease states and the change from active (field) to torpid (cave) physiological states.

## 4. Discussion

The VOC profiles determined from whole-body volatile emissions of individual bats varied widely depending on physiological states, defined by levels of physical activity (active vs. torpid), environment (inside or outside of caves), and WNS-disease status. Volatile emissions from physically active healthy field bats contained 114 total VOCs with a wide range of molecular weights and consisted of 16 major components from at least 8 chemical classes. The diversity of VOCs in emissions from primarily torpid cave bats was substantially reduced with healthy individuals emitting 97 total detectable VOCs and Pd-infected individuals emitting only 72 detectable total VOCs. These emissions included 11 major components from 6 chemical classes for healthy torpid bats and 8 major components from 5 chemical classes in WNS-diseased torpid bats. The greater abundance and diversity of VOC emissions detected in healthy active bats, compared to torpid cave bats, suggest differences in levels of physiological activities. The attenuation of many metabolic pathways that occur as bats enter the torpid state for hibernation explains much of the reduction in VOC emissions from torpid bats compared to active bats. Active bats have additional metabolic pathways operating in association with flying and feeding (muscular) activities which are absent in sedentary, torpid bats that have only diminished basal metabolic activities in operation. Field bats also tend to show greater exposure to outdoor environmental pollutants and agricultural pesticides.

The most common VOC type collected in air samples from all sample types were in the alkane chemical class. Alkanes accounted for a large majority of VOCs detected in volatile emissions from healthy field bats (25%), healthy cave bats (45%), and WNS-diseased bats (50%), respectively. Among the alkane VOC emissions, heptane was unique to active field bats, while 2-methylheptane was unique to healthy cave bats. Three alkanes, (5-ethylnonane, 2-methyldecane, 3-methyldecane), were produced in common within VOC emissions from all three groups, but infected bats had much lower emissions of these three alkanes than healthy bats from both field and cave locations. Undecane, found only in volatile emissions from torpid cave bats, was released by healthy bats at twice the levels of Pd-infected bats.

Most alkanes detected among all samples were either methylated or ethylated alkanes. There is some indication that the degree of methylation in alkane emissions in mammals may be an indication or measure of host immune activity. Higher emissions of methylated alkanes have been associated with presence of mammalian immune responses to diseases. Lawal et al. [52] studied VOC signatures from volatile emissions derived from the co-culture of lung epithelial cell line with bacterial pathogen *Pseudomonas aeruginosa* and found several alkanes associated with immune responses. Yang et al. [53] found that lipid peroxidation-induced pentane and C5–C7 methylated alkanes constituted a specific fingerprint in the breath of pneumoconiosis patients. If induction of alkane emissions as an indicator of immune operation applies to similar physiological activities in Pd-infected bats, the higher emissions of methylated and ethylated alkanes in active bats might provide a chemical indicator of greater immune activity operating in healthy field bats than are measured in torpid WNS-diseased bats with altered immune responses.

Alkanes and methylated alkanes also have been associated with oxidative stress and lipid peroxidation [54,55,56]. Moore et al. [57] provided evidence that oxidative stress was a significant factor contributing to WNS-associated mortality. Lawal et al. [52] found several alkanes including decane, hexane, octane and cyclohexane that were elevated when epithelial cells were exposed to oxidative stress. Phillips et al. [55] developed the Breath Methylated Alkane Contour (BMAC) 3-d plot of lung alveolar C_4_–C_20_ mono-methylated alkanes produced by lipid peroxidation (oxidative stress) which could be used as a model to study host responses to certain diseases involving oxidative stress.

Acetone (propan-2-one) derived from fatty acid oxidation can be converted in the liver to form glucose for respiration or stored as glycogen. The rate of conversion of fat to carbohydrate (fatty acid oxidation → acetoacetate → acetone → glucose) normally is determined by levels of physical activity in healthy bats in the absence of starvation. Serum acetone rises proportionally to intensity of physical activity and peaks 15–30 min after cessation of exercise [58]. This explains why acetone (Peak 4, RT = 17.9) was detected in VOC emissions of field bats (shortly after capture) but absent in torpid cave bats. At low acetone concentrations (under normal physiologic situations), acetone is converted to glucose, but at high concentrations (hyperketonemia), acetone is mainly converted to acetate, resulting in ketoacidosis. Torpid cave bats have low metabolic rates and consume fat reserves slowly despite possible starvation conditions. Normally, serum acetone levels in conscious bats in a state of starvation would be expected to be elevated, resulting in higher acetone emissions from exhaled breath. However, Pd-infected bats in torpor, even in advanced stages of starvation, apparently have sufficient metabolic rates (although attenuated due to torpor) to consume serum glucose (generated from acetone via fat conversion), preventing accumulation of serum acetone and release in the breath. Bats with WNS have higher torpid metabolic rates [59]. Warnecke et al. [12] found low serum glucose levels (~70 mg/dl) in WNS-diseased little brown bats (*M. lucifugus*) compared to healthy controls (>100 mg/dl). They concluded that hypoglycemia and ketoacidosis were possible causes of death. This may partially explain why acetone levels were not detected in volatile emissions from Pd-infected cave bats. Acetone concentrations in exhaled breath of mammals previously have been shown to correlate strongly with acetone and glucose concentrations in blood [60].

Three ketone bodies, (acetone, acetoacetate, and 3-β-hydroxybutyrate), are produced as alternative energy sources within the mammalian liver when glucose is less available, such as during starvation periods. Extracave bats involved in prolonged active exercise (e.g., when feeding and flying), tend to have low serum glucose levels and elevated ketone bodies in their blood. Energy shortages cause the liver to break down fats at a higher rate and increase production of serum ketone bodies to supply metabolic needs while bats are in starvation states with low energy reserves. Among the three ketone bodies, acetone is the most volatile and thus most abundant in the exhaled breath bats, compared to the other two VOCs, during starvation. One additional small molecular weight ketone (Acetol or 1-hydroxy-2-propanone), a possible metabolic derivative of acetone), was also found in volatile emissions from active field bats that were absent in cave bats.

We found more than a dozen agricultural pesticides from several chemical classes within whole-body emissions, including highly toxic organophosphate and carbamate insecticides. The detection of pesticide VOCs within volatile emissions has significance as major contributing factors to exacerbate the negative pathophysiological effects of WNS. Bat exposure to pesticides has been shown to negatively affect bat fat reserves through increases in metabolic respiration that accelerates fat consumption, reducing fat reserves of starving bats needed for winter survival [61]. As pesticide-contaminated fats are consumed, pesticides are remobilized, causing neurotoxic effects (by organophosphates carbamates) that may contribute to increased mortality [62].

Pesticide bioaccumulation within body fats have the potential to affect immune response, enzyme activity, reproduction, and contribute to detrimental effects and increased disease-associated mortality [62]. Some reports have recorded sublethal effects of organophosphates in mammals include interference in normal behavior, oxidative stress, abnormal metabolic and endocrine changes, and reduction in the effectiveness of immune system functions and body thermoregulation [61,63,64,65]. Chemical contaminants may have synergistic or additive detrimental effects on disease mechanisms when WNS-diseased bats undergo unusually frequent arousals from hibernation that accelerate depletion of fat reserves [11,13].

Insectivorous bats receive most pesticide exposure from consuming contaminated insects, but pesticide absorption through the skin is also possible due to their large surface area to body weight ratio. Bats store pesticides primarily in fat, brain, and liver tissues [66,67,68]. Bats are capable of metabolically degrading many pesticides in their body prior to fat storage. However, the metabolic products derived from pesticide degradation may also be bioaccumulated within fat reserves, inducing immunotoxicity and reproductive failure [69].

We discovered five active field (AF) VOC biomarkers, (tentatively identified as acetaldehyde, acetol, 2-methyl hexane, 4-ethyl hexadecane, and heptadecanal), common to active summer bats which were mostly absent in torpid bats except for a few healthy cave bats. These AF biomarkers consisted of two aldehydes, a ketone, and an alkane. We propose several possible explanations for the occurrence of AF biomarkers in some healthy torpid cave bats. Southern bats, unlike northern species of insectivorous bats, commonly fly out of the cave to feed during warmer winter periods, detected by cave bats via lower cave barometric pressure. This winter feasting activity requires energetically costly arousals that consume some fat reserves, but presumably result in a net energy gain if sufficient insects are consumed during these feeding bouts [70,71]. Some cave bats regularly fly to new locations within caves to find better conditions for hibernating sites (with different temperature or humidity conditions). Regular arousals during torpor also are an important means for stimulating the reactivation of parts of the immune system in healthy bats which generally have greater fat reserves [72]. This mechanism of arousal for stimulating the immune system in healthy bats may not be activated in WNS-diseased bats (at a lower level of consciousness), precluding emissions of AF biomarkers. Nevertheless, the often more frequent arousals in Pd-infected bats, due to irritation caused by Pd attacks, could involve a different physiological mechanism of arousal that do not activate AF-biomarker emissions. AF biomarkers provide indications of significant metabolic activity associated with physical activity.

Two torpor-specific (T) VOC biomarkers, tentatively identified as 3-hexanol and ethylcyclohexane, were only found in torpid cave bats, and absent in active field bats. The T-biomarkers include an alcohol and a cycloalkane, respectively. The T-biomarkers provide metabolic indications that bats are in a torpid state at the specific time the air samples were collected. These VOC emissions presumably are released from torpor-specific metabolic pathways that initiate and operate only when bats enter torpor. Metabolic activities that occur when bats have consciousness or are engaged in physical activity, resulting in release of AF-biomarker VOCs, are predominantly shut down and replaced by T-biomarker emissions. Bats undergo physiological changes from summer active to winter torpid states to reduce respiration rates, circulatory activities, and catabolic pathways primarily to conserve energy [41,42,43]. Thus, emissions of VOCs from bats can change dramatically over time as their physiological states are altered by environment cues, such as ambient conditions outside or inside of caves, or internal hormonal mechanisms controlling entry into torpor and arousal periods. Different types of volatile biomarkers may be monitored over time to detect internal physiological changes associated with specific types of metabolic activities occurring at different times when VOC emission are analyzed.

Four metabolomic Health (H)-associated VOC biomarkers, (including compounds tentatively identified as ethanol, 4-ethylheptane, glycerol, and γ-decalactone), were found to be emitted at significantly higher levels in healthy bats, both in active field and healthy cave bats, but were released at much lower levels or not at all in WNS-diseased bats. The H-biomarkers were from alcohol, alkane, polyol, and lactone chemical classes. Detection of H-biomarkers (at specific levels) potentially may provide indications of the relative health state of bats (healthy, in decline, or diseased), based on H-biomarker emission rates, useful for quickly monitoring bat clusters at different locations in caves and to assess and estimate the health state of an entire cave population, through suitable air-sampling surveys in the vicinity of bats, throughout the cave. Additional research is warranted to confirm the identities and chemical structures of putatively identified bat metabolites defined as specific biomarker types in this paper.

Two additional metabolomic biomarkers, designated as conscious activity (CA) VOC biomarkers, were found at emission levels that appeared to correspond to relative physiological activities associated with levels or duration of consciousness. CA-biomarkers emissions were highest in active field bats and considerably lower in torpid field bats. The CA-biomarkers do not appear to be related to physiological activities associated with vigorous physical activities (such as flying), but are more associated with sedentary awake activities such as cleaning or grooming off activities which are conscious activities more commonly practiced by active field bats or bats that have recently entered a cave (and have not yet initiated torpor), and less common in torpid cave bats that engage in grooming activities only for brief intermittent periods during infrequent prolonged arousals. The release of CA-biomarkers in cave bats also may possibly be correlated with activation of major histocompatibility complex (MHC) immune responses when bats are awake, involving cytokine production, that have been indicated as adaptive immunity to WNS in certain European bat species developing long-term resistance, but may not yet be fully or effectively operating in North American bat species due to only recent occurrence of this disease [57,73,74].

McGuire et al. [59] provided direct evidence that heightened energy expenditures during torpor and higher evaporative water loss independently contribute to WNS pathophysiology. Pd-infected little brown bats (*Myotis lucifugus*) have been observed to have significantly higher torpid metabolic rates and evaporative water loss compared to uninfected controls [59,75]. In addition, the downregulation of immune functions during hibernation allows the Pd-pathogen to invade skin tissues without confronting a strong immune response [76]. Bats with more severe WNS symptoms typically exhibit more frequent arousals [11]. Fuller et al. [77] found little brown bats expressed relatively shallow torpor bouts during intensive healing at the end of hibernation and face a severe energetic imbalance during early recovery from WNS.

The 3-d PCA results, based on MOS e-nose sensor array analysis of VOC emissions, indicated significant differences in VOC composition of gaseous emissions from active field, healthy cave, and WNS-diseased bats. Tentative identifications of individual VOCs detected within volatile emissions using dual-column GC with Kovats Retention Indices and RI values confirmed differences in composition of each sample type. Differences in chemical relatedness of VOCs were further confirmed by PDI values, derived from this quantitative statistical indicator, providing precise percentage values of chemical relatedness between of sample types through pairwise comparisons in all possible combinations. These differences in VOC emissions provide strong evidence of differences in physiological states of active vs. torpid bats caused by metabolic changes that occur in response to a multitude of factors including levels of physical activity, consciousness, environmental factors, and health conditions associated with Pd-infections, severity of WNS-disease development, and related immune responses.

Bat VOC emissions from whole-body air sampling include emissions from the skin, hair, and the breath (mouth and lungs). Additional VOCs also could be emitted from urine and feces excretions with various microflora and resident microbes on the skin as well as the Pd-pathogen and VOCs derived from pathogenesis. Skin and hair VOC alterations, due to chemical exposure to biological and chemical contaminants in field and or caves, also are possible. Any of these possible variable sources of VOCs could potentially alter the VOC profiles of each samples type to confound the results. However, Lutz et al. [78] compared the gut microbiomes of insectivorous and frugivorous bats from multiple anatomical sites and geographic localities and concluded that gut, oral, and skin microbiota of bats are shaped predominantly by ecological factors and do not exhibit the same degree of phylosymbiosis observed in other mammals. Diet and host phylogeny primarily drive the taxonomic and functional contents of gut microbiome for most mammals, but there is little correlation between diet and variations in gut microbiome phylogeny in bats [79]. Our PCA aroma maps results showed tight clustering of sample types within data plot clusters despite bat samplings from multiple cave sources, indicating that VOC profiles within each sample type were not significantly altered by variable VOC sources that were possible from many different caves and locations.

Virulence factors associated with WNS pathogenesis contribute to changes in bat host metabolic pathways which likely result in changes in VOC emissions that could be identified in subsequent studies. The most important WNS virulence factors produced directly by the Pd-pathogen include extracellular proteases that digest bat skin tissues and riboflavin (vitamin B_2_) that contributes to oxidative stress, cytokine storms, and IRIS-related effects [9,74,80,81].

## 5. Conclusions

The cumulative results presented here suggest that new e-nose EAD-technologies, based on GC/E-nose dual-technology VOC-detection capabilities and analyses of physiological states by VOC emissions, provide additional information and a new noninvasive alternative for early assessment of Pd-infection and WNS-disease status, avoiding the semi-invasive and tenuous early-detection capabilities of qPCR that require tactile swabs from external bat skin surfaces. This approach also allows the opportunity to monitor gradual metabolic changes (indicative of complex physiological alterations) that occur in transitions of host metabolic pathways (physiological states) from healthy to advanced diseased states and from active bat activities to torpid states during winter hibernation.

Additional research is needed to further explore the gradual changes in physiological states of bats from when they first enter caves in late autumn or early winter, acquire Pd-infections within the cave, and transition to increasingly severe WNS-disease states throughout the winter during hibernation. The effects of gradual physiological changes that occur due to WNS-associated pathogenesis could be better elucidated with additional pathophysiological analysis of VOC emissions from bats in different stages of WNS disease develop over time within winter hibernacula. More research also is needed to determine the originating sources of individual VOCs occurring in whole-body emissions.

## Figures and Tables

**Table 1:** Gas chromatographic data indicating tentative identities of whole-body VOC-metabolite emissions associated with major peaks derived from active healthy, field tricolored bats (n = 4, N = 8).

Peak	RT ^1^	Peak Area	KRI-v ^2^	Tentative Identity	CAS No. ^3^	KRI-t ^4^	RI Range ^5^	Chemical Class
1	13.6	646.3	414	Trimethylamine	75-50-3	425	82.5–91.0	Amine
2	14.3	127.4	431	Acetaldehyde	75-07-0	429	46.6–93.4	Aldehyde
				Methanol	67-56-1	425	45.1–94.1	Alcohol
3	15.3	335.5	449	Ethanol	64-17-5	449	20.8–89.9	Alcohol
4	17.9	693.1	512	Acetone (propan-2-one)	67-64-1	498	76.9–94.4	Ketone
				Propanal	123-38-6	499	77.7–92.9	Aldehyde
				Ethanethiol	75-08-1	516	77.4–95.7	Thiol
5	21.3	516.0	590	S(+)-2-butanol	78-92-2	591	92.4–92.9	Alcohol
				3-methylpentane	96-14-0	579	79.7–96.2	Alkane
6	26.5	161.3	656	Acetol (1-hydroxy-2-propanone)	116-09-6	655	77.7–80.2	Ketone
				3-methylfuran	930-27-8	630	75.3–90.0	Furan
7	30.0	614.4	697	Heptane	142-82-5	700	88.7–90.9	Alkane
				3-ethylpentane	617-78-7	685	92.2–94.9	Alkane
8	50.4	1543.3	882	3-mercapto-4-methyl- 2-pentanone	75832-79-0	883	88.4–97.4	Ketone
				2-methylbutanoic acid	116-53-0	872	82.4–98.2	Carboxylic acid
				2-furanmethanol	98-00-0	860	81.9–97.6	Alcohol
9	58.9	2491.0	982	Phenol	108-95-2	986	93.7–96.1	Benzene alcohol
				Dimethylethylbenzene	98-06-6	990	92.7–94.5	Benzene derivative
10	62.1	1959.5	1027	Limonene	138-86-3	1029	96.7–98.3	Cyclic monoterpene
				1-heptanethiol	1639-09-4	1021	96.9–98.5	Alkanethiol
11	63.3	3181.6	1045	5-ethylnonane	17302-12-4	1051	87.0–97.8	Alkane
				1-methyl-4-isopropenyl-1-cyclohexene	138-86-3	1030	92.8–96.7	Cyclohexene, monoterpene
12	64.8	4649.0	1067	2-methyldecane	6975-98-0	1064	94.8–99.7	Alkane
				4-methyldecane	2847-72-5	1060	91.3–99.0	Alkane
13	65.9	1979.3	1084	3-methyldecane	13151-34-3	1071	89.8–97.4	Alkane
				Undecane	1120-21-4	1100	90.6–95.9	Alkane
14	82.8	532.5	1402	δ-nonalactone	3301-94-8	1404	78.3–96.9	Lactone
				Methyl eugenol	93-15-2	1404	79.2–93.1	Phenyl propene
15	95.0	975.5	1688	2-pentadecanol	1653-34-5	1710	94.33	Alcohol
				Butyl cinnamate	538-65-8	1702	94.62	Ester
16	104.9	137.9	1919	Heptadecanal	629-90-3	1920	85.7–96.4	Aldehyde
				Pentadecyl acetate	629-58-3	1907	68.2–97.7	Ester

^1^ Retention times (to 0.01 s, s.d. = 0.02) of VOCs present in major-peak whole-body emissions detected with a 10 m DB-5 column using GC-analysis parameters specified previously. ^2^ KRI-v = Kovats Retention Index known values for specific VOC metabolites, represented by an individual peak and retention time for a 10 m DB-5 column using 11-alkane (C7–C17) analytical reference-standard calibration. ^3^ CAS number = Chemical Abstracts Service (CAS) Registry Number, unique numerical identifier. ^4^ KRI-t = Kovats Retention Index for tentative identify for compounds; indicated as most probable identity based on closest KRI-values. ^5^ RI = Relevance Index, indicating percentage probability of identity match, based on Kovats values for the specified tentative-identity reference compounds, determined from dual-column data derived from 10 m DB-5 and DB-1701 columns with analytical reference standards; NA = not available (due to limited data from all samples).

**Table 2:** Gas chromatographic data indicating the tentative identities of whole-body VOC-metabolite emissions associated with major peaks derived from inactive (mostly torpid) healthy, intracave tricolored bats (n = 15, N = 30).

Peak	RT ^1^	Peak Area	KRI-v ^2^	Tentative Identity	CAS No. ^3^	KRI-t ^4^	RI Range ^5^	Chemical Class
1	15.3	392.9	453	Propenal	107-02-8	450	60.0–94.4	Aldehyde
				Methanethiol	74-93-1	449	60.4–89.3	Thiol
				2-methylbutane	78-78-4	464	60.0–89.3	Alkane
2	19.6	931.1	551	t-butylmethylether	1634-04-4	546	77.5–97.2	Ether
				2-methylpentane	107-83-5	560	60.3–96.0	Alkane
				Cyclopentane	287-92-3	567	76.2–95.6	Cycloalkane
3	37.1	1840.7	758	2-methylheptane	592-27-8	765	94.8–98.1	Alkane
				4-methylheptane	589-53-7	767	90.3–98.2	Alkane
4	50.4	2806.1	882	3-mercapto-4-methyl- 2-pentanone	75832-79-0	883	92.2–99.0	Ketone
				2-methylbutanoic acid	116-53-0	872	70.3–96.9	Carboxylic acid
				Pentanoic acid	109-52-4	903	88.9–95.5	Carboxylic acid
5	58.9	1413.8	982	Phenol	108-95-2	986	88.0–99.2	Benzene alcohol
				Dimethylethylbenzene	98-06-6	990	91.7–96.5	Benzene deriv.
6	63.3	3430.5	1045	5-ethylnonane	17302-12-4	1051	92.2–96.7	Alkane
				4-ethylnonane	5911-05-7	1053	91.8–96.9	Alkane
7	64.8	4423.9	1067	2-methyldecane	6975-98-0	1064	92.2–98.8	Alkane
				4-methyldecane	2847-72-5	1060	91.8–98.1	Alkane
				5-methyldecane	13151-35-4	1058	91.2–97.5	Alkane
8	66.0	1717.6	1084	3-methyldecane	13151-34-3	1071	88.4–96.7	Alkane
				γ-terpinene	99-85-4	1060	88.0–96.3	Monoterpene
9	67.2	622.6	1103	Undecane	1120-21-4	1100	86.0–98.0	Alkane
				α-terpinolene	586-62-9	1088	85.3–98.0	Menthane monoterpenoid
				2-isopropyl-3-methoxypyrazine	25773-40-4	1097	86.0–97.4	Pyrazine
10	82.8	446.6	1402	δ-nonalactone	3301-94-8	1404	69.7–92.6	Lactone
				Methyl eugenol	93-15-2	1404	70.2–93.5	Phenyl propene
				Histamine	51-45-6	1415	13.1–90.9	Histamine
11	95.0	384.6	1688	δ-dodecalactone	713-95-1	1715	93.3–97.3	Lactone
				Dodecan-4-olide	18679-18-0	1677	94.6–98.7	Lactone

^1^ Retention times (to 0.01 s, s.d. = 0.02) of VOCs present in major-peak whole-body emissions detected with a 10 m DB-5 column using GC-analysis parameters specified previously. ^2^ KRI-v = Kovats Retention Index for specific volatile metabolite represented by the individual peak and retention time for a 10 m DB-5 column using 11-alkane (C7–C17) analytical reference-standard calibration. ^3^ CAS number = Chemical Abstracts Service (CAS) Registry Number, unique numerical identifier. ^4^ KRI-t = Kovats Retention Index for tentative identify for compounds; indicated as most probable identity based on closest KRI-values. ^5^ RI = Relevance Index, indicating percentage probability of identity match, based on Kovats values for the specified tentative-identity reference compounds, determined from dual-column data derived from 10 m DB-5 and DB-1701 columns with analytical reference standards; NA = not available (due to limited data from all samples).

**Table 3:** Gas chromatographic data indicating the tentative identities of whole-body VOC-metabolite emissions associated with major peaks derived from inactive Pd-infected (symptomatic WNS-diseased), intracave tricolored bats (n = 9, N = 18).

Peak	RT ^1^	Peak Area	KRI-v ^2^	Tentative Identity	CAS No. ^3^	KRI-t ^4^	RI Range ^5^	Chemical Class
1	50.4	1129.9	882	3-mercapto-4-methyl- 2-pentanone	75832-79-0	883	83.5–92.7	Ketone
				2-methylbutanoic acid	116-53-0	872	91.2–96.6	Carboxylic acid
2	58.9	904.3	982	Phenol	108-95-2	986	93.3–99.0	Benzene alcohol
				1-octen-3-one	4312-99-6	979	87.4–98.6	Ketone
				Dimethyl trisulfide	3658-80-8	970	93.1–97.7	Sulfide
3	63.3	1468.2	1045	5-ethylnonane	17302-12-4	1051	89.6–94.2	Alkane
				4-ethylnonane	45911-05-7	1053	89.2–93.8	Alkane
4	64.8	1964.9	1067	2-methyldecane	6975-98-0	1064	89.5–99.1	Alkane
				4-methyldecane	2847-72-5	1060	88.9–98.5	Alkane
				5-methyldecane	13151-35-4	1058	88.3–97.8	Alkane
5	66.0	758.2	1084	3-methyldecane	13151-34-3	1071	91.2–95.8	Alkane
				Butylbenzene	104-51-8	1058	85.8–93.9	Benzene derivative
6	67.2	310.1	1103	Undecane	1120-21-4	1100	80.3–96.0	Alkane
				cis-decalin	493-01-6	1106	78.6–97.0	Bicyclic HC
7	77.5	221.6	1292	Hexyl pentanoate	1117-59-5	1293	98.37	Ester
				1-p-menthen-8-thiol	71159-90-5	1285	62.3–98.2	Thiol
				Tridecane	629-50-5	1300	71.5–98.6	Alkane
8	82.8	454.6	1405	δ-nonalactone	3301-94-8	1404	75.2–96.0	Lactone
				Methyl eugenol	93-15-2	1404	75.5–96.5	Phenyl propene

^1^ Retention times (to 0.01 s, s.d. = 0.02) of VOCs present in major-peak whole-body emissions detected with a 10 m DB-5 column using GC-analysis parameters specified previously. ^2^ KRI-v = Kovats Retention Index for specific volatile metabolite represented by the individual peak and retention time for a 10 m DB-5 column using 11-alkane (C7–C17) analytical reference-standard calibration. ^3^ CAS number = Chemical Abstracts Service (CAS) Registry Number, unique numerical identifier. ^4^ KRI-t = Kovats Retention Index for tentative identify for compounds; indicated as most probable identity based on closest KRI-values. ^5^ RI = Relevance Index, indicating percentage probability of identity match, based on Kovats values for the specified tentative-identity reference compounds, determined from dual-column data derived from 10 m DB-5 and DB-1701 columns with analytical reference standards; NA = not available (due to limited data from all samples).

**Table 4:** Summary of major and minor whole-body VOC emissions from healthy active extracave (field) bats, healthy inactive intracave bats, and WNS-diseased intracave tricolored bats.

VOC Chemical Classes ^1^	Healthy Field ^2^	Healthy Cave ^2^	Infected Cave ^2^
Alcohols	3	-	-
Aldehydes	2	1	-
Alkanes	4	5	4
Amines	1	-	-
Benzene alcohols	1	1	1
Esters	-	-	1
Ethers	-	1	-
Ketones	3	1	1
Lactones	1	2	1
Terpenes	1	-	-
Totals:			
Major VOC peaks	**16**	**11**	**8**
Minor VOC peaks	**98**	**86**	**64**
All VOC peaks	**114**	**97**	**72**

^1^ Chemical classes of VOCs detected in whole-body emissions from bats based on the primary functional groups of individual compounds present in the sample headspace. ^2^ Tricolored bat whole-body air sample types (aroma classes): Healthy field bats = physically active foraging bats with full-range of metabolic activities; Healthy cave bat = physically inactive, Pd-uninfected, torpid bats with greatly reduced physiological activity; Pd-infected cave bats = Pd-infected, WNS-symptomatic torpid bats with reduced physiologically activity, but more frequent arousal episodes due to dermatophytic, Pd-associated irritation.

**Table 5:** Agricultural pesticides present in whole-body VOC-emissions from healthy active extracave (field) bats, healthy inactive intracave bats, and WNS-diseased intracave tricolored bats.

					GC Peak Areas Range/(no. Bats) ^3^ *P. subflavus* Bat Type		
RT ^1^	Pesticides ^2^	RI Range	Chemical Classes	Pesticide Types	Healthy Field	Healthy Cave	Infected Cave
75.6	Dichlorvos	86.0–98.5	Organophosphate	Insecticide	-	80–145 (7)	64–113 (2)
88.0	Molinate	63.1–97.2	Thiocarbamate	Herbicide	137–552 (1)	75–95(2)	-
91.8	Demeton-O	88.4–94.8	Phosphorothioate	Insecticide	140–385 (1)	59–60 (2)	-
93.6	Dicrotophos	91.9–97.7	Organophosphate	Insecticide	51–235 (2)	66–107(4)	-
	Carbanilic acid, isopropyl ester	74.4–99.4	Carbamate ester	Herbicide	161–235 (1)	51–93 (6)	-
	Sulfotep	91.4–94.0	Organothiophosphate	Insecticide	-	-	70–104 (2)
95.0	Atraton	53.7–96.9	Diaminotriazine	Herbicide	-	64–665 (2)	112–160 (3)
	Phorate	55.4–92.3	Organothiophosphate	Insecticide	70–92 (1)	409–470 (3)	83–428 (3)
96.8	Atrazine	71.2–97.8	Triazine	Herbicide	-	55–58 (1)	-
	trans-Diallate	46.9–97.2	Thiocarbamate	Herbicide	-	56–264 (5)	60–86(3)
	Dimethoate	59.6–81.4	Organophosphate	Insecticide	871–1521 (1)	56–81 (1)	56–68 (3)
98.2	Propazine	77.9–97.0	Chloro-s-triazine	Herbicide	51–54 (1)	-	-
	Delnav II	78.4–98.3	Organophosphate	Insecticide	-	57–174 (2)	-

^1^ Retention times (to 0.01 s, s.d. = 0.02) for pesticide VOCs present in minor-peak whole-body emissions detected with a 10 m DB-5 column using GC-analysis parameters specified previously. ^2^ Pesticide common chemical names. ^3^ Peak areas are represented as ranges of areas (under each chromatogram peak curve) for the given number of bats (indicated in parentheses).

**Table 6:** Biomarker metabolites of tricolored bats, indicative of physiological states based on whole-body minor-peak VOC-emissions, associated with healthy active extracave (field) bats (n = 4, N = 8), healthy inactive intracave bats (n = 15, N = 30), and WNS-diseased intracave bats n = 9, N = 18).

			Tentative Identity of *P. subflavus* VOC Biomarkers					
Peak	RT ^1^	KRI-v ^2^	Healthy Field	Healthy Cave	Pd-Infected Cave	CAS no. ^3^	RI Range ^4^	Chemical Class
1	14.3	431	Acetaldehyde	-	-	75-07-0	46.6–93.4	Aldehyde
			Methanol	-	-	67-56-1	45.1–94.1	Alcohol
2	15.3	449	Ethanol	Propenal	-	64-17-5 123-38-6	20.8–89.9 60.0–94.4	Alcohol Aldehyde
			-	Methanethiol	-	74-93-1	60.4–89.3	Thiol
			-	2-Methylbutane	-	78-78-4	60.0–89.3	Alkane
3	26.5	656	Acetol	-	-	116-09-6	77.7–80.2	Ketone
			3-methylfuran	-	-	930-27-8	75.3–90.0	Furan
4	27.6	667	2-methyl hexane	-	-	591-76-4	61.1–74.4	Alkane
			Cyclohexane	-	-	110-82-7	61.4–73.8	Cycloalkane
5	42.7	810	-	3-hexanol	Propyl propanoate	623-37-0 106-36-5	78.5–92.7 93.8–96.7	Alcohol Ester
			-	2-hexanol	-	626-93-7	78.1–93.2	Alcohol
6	45.7	839	-	Ethylcyclohexane	Ethylcyclohexane	1678-91-7	49.8–96.7	Cycloalkane
			-	Propylcyclo-pentane	Propylcyclo-pentane	2040-96-2	50.3–97.9	Cycloalkane
7	48.1	862	4-ethylheptane	4-ethylheptane	Ethyl benzene	2216-32-2 100-41-4	56.0–98.0 65.3–95.7	Alkane Benzene deriv.
8	56.3	948	Glycerol	Aniline	-	56-81-5 62-53-3	82.5–92.2 77.2–93.9	Polyol Analine
			δ-valerolactone	3-methyl-3-sulfanylbutanol-1-ol	-	542-28-9 34300-94-2	64.7–84.6 75.9–93.4	Lactone Alcohol
9	60.7	1006	2-(2-ethoxyethoxy) ethanol	2,4-heptadienal	2-(2-ethoxyethoxy) ethanol	111-90-0 5910-85-0	60.8–96.5 78.68–98.8	Alcohol Aldehyde
			2,4-heptadienal (E,E)-	-	2,4-heptadienal (E,Z)	4313-03-5 4313-02-4	60.6–96.7 78.78–99.27	Aldehydes
10	72.4	1192	Ethyl octanoate	2-decanone, 3-decanol	5-ethyl-3-hydroxy-4-methyl-2(5H)-furanone	106-32-1 693-54-9 1565-81-7 698-10-2	94.2–95.5 94.8 94.5 75.4–98.2	Ester Ketone Alcohol Furanone
			Z-3-hexen-1-ol, butanoate	-	2-pentyl-pyridine	16491-36-4 2294-76-0	94.55–95.3 77.4–96.9	Ester Pyridine
11	86.2	1479	γ-decalactone	γ-decalactone	2-methyl-tetradecane	706-14-9 1560-95-8	67.5–96.1 65.7–96.4	Ketone Alkane
			4-methyl-tetradecane	-	4-methyl-tetradecane	25117-24-2	65.7–93.5	Alkane
12	98.6	1769	4-ethylhexadecane	-	-	NA	91.0–97.9	Alkane
			7-methylhepta-decane	-	-	20959-33-5	91.0–97.9	Alkane
13	104.9	1919	Heptadecanal	-	-	629-90-3	85.7–96.4	Aldehyde
			Pentadecyl acetate	-	-	629-58-3	68.2–97.7	Ester

^1^ Retention times (to 0.01 s, s.d. = 0.02) of VOCs present in major-peak whole-body emissions detected with a 10 m DB-5 column using GC-analysis parameters specified previously. ^2^ KRI-v = Kovats Retention Index for specific volatile metabolite represented by the individual peak and retention time for a 10 m DB-5 column using 11-alkane (C7–C17) analytical reference-standard calibration. ^3^ CAS number = Chemical Abstracts Service (CAS) Registry Number, unique numerical identifier, na = not available. ^4^ RI = Relevance Index, indicating percentage probability of identity match, based on Kovats values for the specified tentative-identity reference compounds, determined from dual-column data derived from 10 m DB-5 and DB-1701 columns with analytical reference standards; NA = not available (due to limited data from all samples).

**Table 7:** VOC biomarkers types identified from whole-body VOC-emissions from healthy active extracave (field) bats, healthy inactive intracave bats, and WNS-diseased intracave tricolored bats.

			Mean GC Peak Areas/no. Bats ^4^				
			Handled Bats (Removed from Cave Wall)			Undisturbed Wall Bats ^3^	
Biomarker Type ^1^	No. ^2^	RT ^3^	Healthy Field n = 4	Healthy Cave n = 15	Infected Cave n = 9	Healthy Cave n = 10	Infected Cave n = 10
**Activity specific**							
Active Field	1	14.3	127.4 a	29.8 b	- b	27.6 b	- b
	2	26.5	121.0 a	1.8 b	- b	73.2 ab	- b
	3	27.6	272.0 a	- b	- b	147.3 b	- b
	4	98.6	46.4 a	13.4 b	- b	- b	- b
	5	104.9	120.6 a	56.3 b	2.9 b	- b	2.8 b
Torpor	1	42.7	- c	224.3 a	112.6 bc	292.8 ab	36.9 c
	2	45.7	- b	58.2 a	32.0 ab	121.4 a	5.1 b
**Metabolomic**							
Healthy	1	15.3	335.5 ab	392.9 a	- c	201.5 b	44.9 c
	2	48.1	56.0 a	70.7 a	60.6 ab	71.9 a	2.7 b
	3	56.3	149.9 a	63.7 a	5.9 b	62.9 a	- b
	4	86.2	253.3 a	103.7 ab	61.4 b	108.6 ab	4.0 c
Conscious activity	1	60.7	189.0 a	6.9 b	83.3 a	67.8 b	8.4 b
	2	72.4	772.0 a	19.7 ab	77.5 a	16.8 ab	- b

^1^ Retention times (to 0.01 s, s.d. = 0.02) of VOCs present in whole-body emissions detected with a 10 m DB-5 column using GC-analysis parameters specified previously. ^2^ Biomarker number (in numerical order by retention times). ^3^ Retention times (to 0.01 s) for biomarker VOCs present in minor-peak, whole-body emissions detected with a 10 m DB-5 column using GC-analysis parameters specified previously. ^4^ Peak areas are represented as ranges of areas (under each chromatogram peak curve) for the given number of bats (indicated in parentheses). Data were analyzed using Kruskal–Wallis one-way ANOVA on ranks. Mean GC peak area values within each data row followed by the same letter are not significantly different according to Dunn’s tests at (*p* < 0.01).

**Table 8:** Chemical relatedness between electronic-nose VOC-profiles of *P. subflavus* bat whole-body headspace volatiles analyzed by 3-d PCA with pattern discrimination index.

Aroma Class 1 ^1^	Aroma Class 2 ^1^	PCA Plot Distance ^2^	PDI (%) ^3^
Healthy field bats	Pd-infected cave bats	14,021.01	69.83
	Healthy cave bats	7889.59	37.73
Pd-infected cave bats	Healthy cave bats	11,921.69	77.09

^1^ Bat whole-body air sample types (aroma classes): Healthy field bats = physically active foraging bats with full-range of metabolic activities; Healthy cave bat = physically inactive, Pd-uninfected, torpid bats with greatly reduced physiological activity; Pd-infected cave bats = Pd-infected, WNS-symptomatic torpid bats with reduced physiologically activity, but more frequent arousal episodes due to dermatophytic, Pd-associated irritation. ^2^ PCA distances indicate actual mapping distances between plot centers of aroma class data clusters defined by 3-d principal component analysis (PCA) aromaplot. ^3^ Pattern discrimination index (PDI) values indicate percentage differences in VOC-aroma profile chemical composition determined by pairwise comparisons of aroma class (sample types) based on PCA statistical tests of data derived from the e-nose multisensor array.
